# Adaptive trial for the treatment of depressive symptoms associated with concussion using accelerated intermittent theta burst stimulation (ADEPT): rationale, design and methods

**DOI:** 10.3389/fneur.2025.1605157

**Published:** 2025-06-13

**Authors:** Lindsay M. Oberman, Adriana I. Penafiel, Rachel Dieterich, Cuong T. Phan, Yi-Yu Chou, Dzung L. Pham, Maheen M. Adamson, Christopher E. Hines, Zeynab Rezaee, Zhi-De Deng, Hemant Pal, Sarah H. Lisanby, David L. Brody

**Affiliations:** ^1^National Institute of Mental Health Intramural Research Program, Bethesda, MD, United States; ^2^Henry M. Jackson Foundation for the Advancement of Military Medicine, Uniformed Services University of the Health Sciences, Bethesda, MD, United States; ^3^Palo Alto Veterans Institute for Research, Palo Alto, CA, United States; ^4^Department of Radiology and Bioengineering, Uniformed Services University of the Health Sciences, Bethesda, MD, United States; ^5^WOMEN CoE, VA Palo Alto Healthcare System, Palo Alto, CA, United States; ^6^Department of Neurosurgery, Stanford University School of Medicine, Stanford, CA, United States; ^7^Walter Reed National Military Medical Center, Bethesda, MD, United States; ^8^Department of Behavior Health, William Beaumont Army Medical Center, El Paso, TX, United States; ^9^School of Medicine and Advanced Medical Engineering, Arizona State University, Phoenix, AZ, United States; ^10^Department of Psychiatry and Behavioral Sciences, Duke University, Durham, NC, United States; ^11^Department of Neurology, Uniformed Services University of the Health Sciences, Bethesda, MD, United States; ^12^Department of Radiology and Bioengineering, Military Traumatic Brain Injury Initiative, Uniformed Services University of the Health Sciences, Uniformed Services University, Bethesda, MD, United States

**Keywords:** concussion, major depressive disorder, accelerated intermittent theta burst stimulation, traumatic brain injury, transcranial magnetic stimulation, Bayesian adaptive trial, military, resting state functional MRI

## Abstract

Military service members (SMs) and veterans who have sustained one or more concussions during their service have significantly higher rates of persistent depressive symptoms and suicidality compared to non-injured peers. Despite over 500,000 SMs who have sustained concussions, there are currently no Level I evidence-based treatments for improving depressive symptoms associated with concussion. Accelerated intermittent theta burst stimulation (aiTBS), a specific repetitive transcranial magnetic stimulation (rTMS) protocol, targeted at the dorsolateral prefrontal cortex (DLPFC) has demonstrated efficacy and is cleared for the treatment of Major Depressive Disorder (MDD) by the United States Food and Drug Administration (FDA). The mechanism of action of aiTBS is thought to be via the modulation of functional networks. Herein we outline the design of a multisite, double-blind, randomized, sham-controlled trial of aiTBS for the treatment of depressive symptoms in SMs and veterans with a history of concussion. We present the rationale for this specific design and highlight the potential for personalized neuroimaging-informed parameter determination in this population where brain injuries have resulted in variable structural and functional brain circuitry disruptions. If successful, this project will accelerate solutions to improve the health, well-being, and healthcare of SMs and veterans with depressive symptoms following concussion.

**Clinical trial registration:**

Clinicaltrials.gov, NCT05426967.

## 1 Introduction

In the United States Military Health System (MHS), traumatic brain injury (TBI) has emerged as one of the most prolific medical conditions affecting over 500,000 service members (SMs) between 2000 and 2024 (1). Despite being the most common type of brain injury in SMs (accounting for over 80% of all TBIs) (1), concussion, also known as “mild” TBI (mTBI), frequently remains undiagnosed and untreated. Current or former SMs who have sustained one or more concussions during their service have significantly higher rates of persistent depressive symptoms and suicidality following injury compared to non-injured peers (2–6). Between 2017 and 2021 mental health was the number one reason for a SM to be evacuated from deployment and Major Depressive Disorder (MDD) was the number one diagnosis listed as the reason for evacuation (7). A meta-analysis identified a 2-fold higher risk of suicide among more than 700,000 patients diagnosed with a mild TBI compared to more than 6.2 million individuals who had never experienced a TBI. From 2018 to 2019, 35.5% of TBI-related deaths were categorized as intentional injuries with suicide being the leading cause of TBI-related death (8, 9). The severity of depressive symptoms following concussion is strongly correlated with global disability, rate of recovery, and quality of life (4, 10, 11).

Despite the clear clinical impact of depressive symptoms following concussion, there remains a paucity of evidence-based effective treatments available to SMs and veterans leaving many with significant residual disease burden. There are currently no Level I evidence-based treatments for improving depressive symptoms associated with concussion. Additionally, the standard pharmacological interventions that are often first-line treatments for depression in the general population (e.g., SSRIs) have failed to show efficacy in the context of TBI in randomized controlled trials (12, 13). Furthermore, pharmacotherapy and behavioral therapy may be inaccessible for certain SMs due to operational training/mission requirements, access to care within the MHS, fear of deployment limitation, and even potential removal from the military (14, 15).

The mechanism underlying the presence of depressive symptoms following military concussion is thought to involve unique multifactorial biological and psychosocial contributors. Notably, individual resting state fMRI-based network mapping has revealed different patterns of functional connectivity in patients with TBI plus depression symptoms compared with patients with TBI without depression symptoms or patients with depression symptoms in the absence of TBI (16). Thus, symptom-targeted, neural circuit-based approaches such as repetitive transcranial magnetic stimulation (rTMS) may be more effective than medications for this population. The FDA cleared rTMS for the treatment of adult treatment-resistant MDD based on trials in patients without co-occurring neurological disorders in 2008 (17, 18). Additionally, an aiTBS protocol, “SNT,” was FDA cleared following a 2022 trial for treatment resistant MDD (19). In this trial, SNT, which involves 10 sessions per day of a specific aiTBS protocol over the course of 5 days, resulted in a 79% remission rate based on a Montgomery-Asberg Depression Rating Scale (MADRS) score of less than 10, the remission cutoff. Of particular relevance to concussion, evidence suggests that rTMS may promote reorganization of brain networks which may enhance recovery following injury (20). This finding is also in-line with data supporting the use of TMS in post-stroke recovery. However, historically, a history of concussion (or any TBI) has been exclusionary for rTMS studies; thus, there is a paucity of literature on the safety and efficacy of rTMS in this population.

Consistent with the original FDA label, rTMS for MDD most commonly targets the left hemisphere DLPFC, a key node in the functional pathophysiology thought to underlie depressive symptoms (21). In addition to its role in depression, the DLPFC plays a critical role in emotion regulation, working memory, and executive functioning. These are all impaired in individuals with a history of concussion and shown to improve after rTMS targeted at the DLPFC (22, 23). Most clinical rTMS treatments for MDD occur daily (one rTMS session per day) and are applied using scalp-based targeting of DLPFC. These standard treatments are only modestly effective, even in the general MDD population (17, 24–31). Additionally, although individuals have remarkably variable functional brain circuitry (32), most clinics apply a “one size fits all” scalp-targeted approach, neglecting to account for individual differences. Compounding the variability in functional brain circuitry seen in the general population, those with a history of concussion may have unique disruptions in functional networks and a complex neurobehavioral clinical presentation (16, 33, 34) making standard scalp-based DLPFC targeting even less optimal for this population.

Recent studies indicate that rTMS affects both local excitability in the stimulated brain region and broader functional connectivity across networks. At least some of the clinical effects may be due more to changes in functional connectivity across the brain regions than local effects on the stimulated region itself (35). In recent years, it has become possible to construct a resting-state functional magnetic resonance imaging (rsfMRI)-based connectome map with high test–retest reliability in an individual subject (36, 37). Two recent papers found that clinical efficacy of rTMS for depression was greater when the scalp-targeted stimulation site was incidentally closer to the optimal rsfMRI-derived site (38, 39).

In addition to targeting, another recent optimization is in the efficiency of the stimulation protocol including session duration and inter-session interval. Standard protocols apply stimulation once a day in a 19- or 37-min 10 Hz session. However, a more efficient once a day 3-min iTBS protocol was FDA cleared in August 2018. Most recently, in September 2022, the FDA cleared the “SNT” protocol that combines a proprietary form of individualized rsfMRI-based targeting strategy with an accelerated schedule (10 times per day) (19). Cole and colleagues found that active aiTBS leads to a 52.5% reduction in depressive symptom severity with 86% of the active group responding (≥50% reduction in symptom severity) and 79% remitting (a MADRS score ≤ 10). Those randomized to receive sham aiTBS experienced an 11.1% reduction in symptom severity with 27% responding and 13% of participants experiencing remission following sham aiTBS. This trial was stopped at the planned interim analysis (32 patients) due to the large effect size (Cohen’s d of 1.4) in the prespecified primary outcome measure. This is the largest effect size observed to date for any rTMS protocol. It is not known whether the apparently superior effects of SNT were driven by the high number of treatments (50 total), the advanced rsfMRI targeting, the accelerated schedule, or the specific group of people studied.

Here, we present the protocol for an ongoing multisite, double-blind, randomized, sham-controlled study of aiTBS applied to the DLPFC eight times per day for 1 week (40 sessions) for treatment of depressive symptoms in SMs and veterans with a history of concussion. Participants will be randomized to receive either active rsfMRI-targeted aiTBS, active Beam F3 scalp-targeted aiTBS, or sham (placebo) aiTBS. Our primary outcome measure will be change in MADRS scores from Baseline to Post-aiTBS between groups. We will leverage our controlled study design to improve our understanding of the relative benefit of individualized rsfMRI targeted approaches in this population and the biological mechanisms of aiTBS treatment in this understudied and underserved population by obtaining rsfMRI both at baseline and immediately after the completion of treatment (“pre/post” treatment imaging).

The objectives of our ongoing trial are to: (1) Evaluate the efficacy of aiTBS as a treatment for depressive symptoms in SMs and veterans with a history of concussion as evidenced by a significant difference in the improvement of depressive symptoms in those randomized to either active stimulation arm as compared to those randomized to the sham (placebo) arm; and (2) Determine whether those randomized to individualized rsfMRI targeting experience greater symptom improvement (pre-post change in the MADRS) compared to those randomized to Beam F3 scalp-based targeting. We hypothesize that active aiTBS will lead to a statistically significant and clinically meaningful ≥4 points depressive symptom reduction at the Post-aiTBS MADRS as compared to sham aiTBS. We also hypothesize that the rsfMRI targeted aiTBS will be superior in reducing depressive symptoms compared to scalp-targeted aiTBS.

## 2 Methods and analysis

### 2.1 Study design

**Figure 1:**
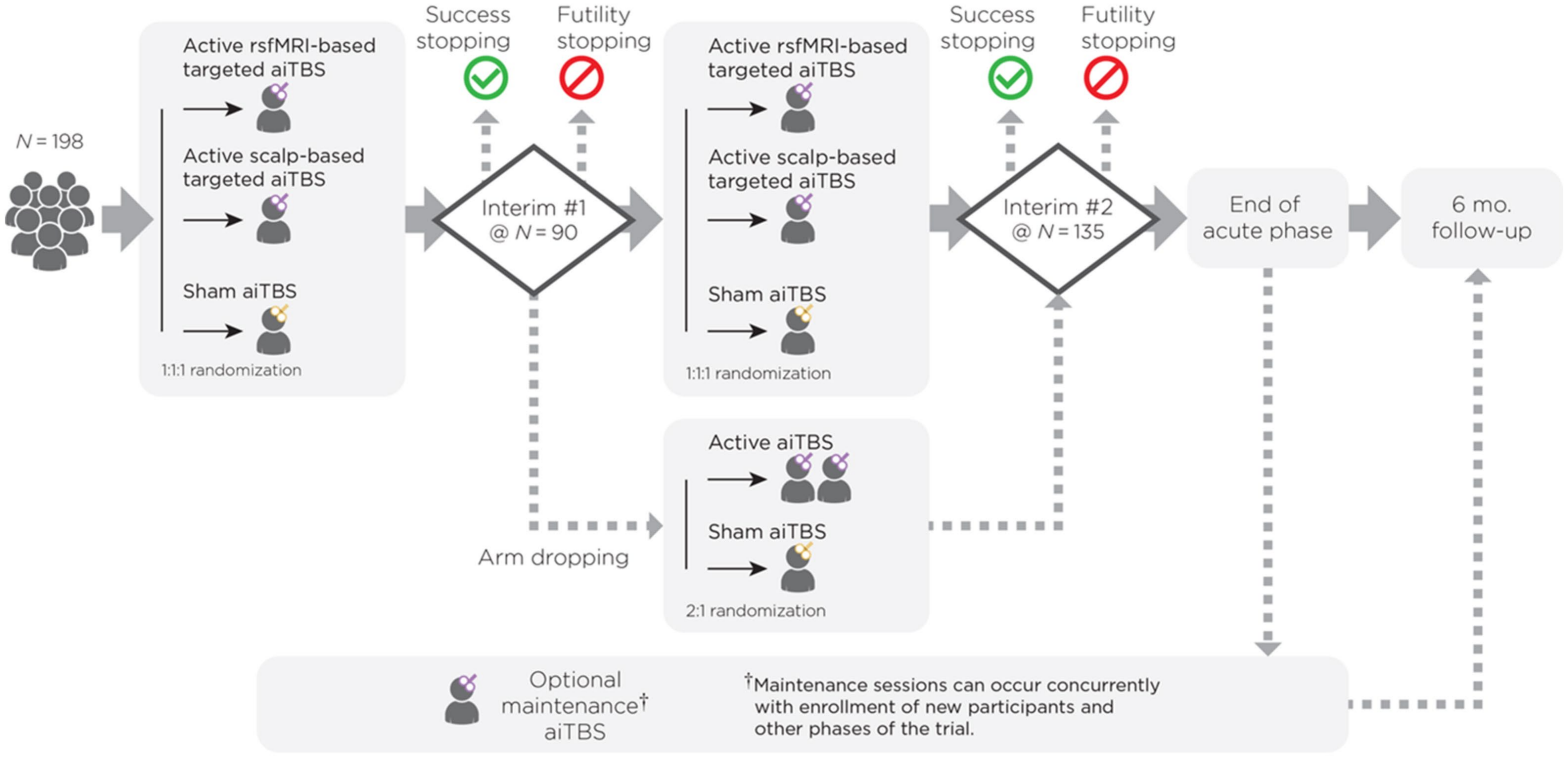
Bayesian adaptive trial schema. The Bayesian adaptive trial for accelerated intermittent theta burst stimulation (aiTBS) with interim analyses at *N* = 90 and *N* = 135. Participants (*N* = 198) are randomized 1:1:1 to (1) active rsfMRI-based targeted aiTBS, (2) active scalp-based (Beam F3) targeted aiTBS, or (3) sham aiTBS. Adaptations include dropping inferior arms (if either active arm has a 60% or greater Bayesian posterior probability of being superior to the other active arm), stopping for futility (if neither active arm has greater than a 60% Bayesian posterior probability of being superior to sham), or stopping for success (if either active arm obtains a 99.75% posterior probability of being superior to sham) if predefined Bayesian probabilities are met. This design ensures efficiency, ethical resource use, and improved participant outcomes while maintaining statistical rigor.

Our ongoing trial uses a Bayesian adaptive, randomized, double-blind, sham-controlled study design (see Figure 1 for Bayesian adaptive trial schema). Bayesian statistical approaches provide a powerful mathematical framework for determining optimal behavior in the face of uncertainty, allow the efficient and transparent integration of complex clinical trial and external data, and offer the ability to learn from the accruing data (40, 41). Specifically, in this trial, the possibility of dropping the less effective active arm may allow more participants to be randomized to the most effective active arm. Initially, eligible participants are randomized between arms in a 1:1:1 fashion to either active rsfMRI-based targeted aiTBS, active Beam F3 scalp-based targeted aiTBS, or sham aiTBS. The TMS system used for this trial has designated blinded active and sham coils identical in appearance, weight, noise emission, and tactile sensation. The coils are identified by a unique label (e.g., “A,” “B,” and “C”). Two coils are active, one is sham. The participants, TMS operator, data collectors, and all other study personnel involved in the final data analysis are blinded to the targeting method and coil (active or sham) variable.

### 2.2 Participants

The study sample includes up to 198 male and female current or former SMs, eligible for care at Department of Defense (DoD) or Veterans Administration (VA) facilities, between the ages of 18 and 55, with a history of concussion and the presence of depressive symptoms. Depending on the results of the interim analyses, the actual sample size may differ and is flexible between 99 and 198 participants. We also anticipate 10% attrition between consent and first treatment thus accounting for 90–180 participants with primary outcome measures for analysis.

The study is being conducted at two military treatment facilities (MTF) [William Beaumont Army Medical Center (WBAMC) and Alexander *T. Augusta* Military Medical Center (ATAMMC) and one VA Medical Center (VAMC) (VA Palo Alto)]. Interested participants are initially provided information about the study and prescreened for eligibility based on inclusion and exclusion criteria. This includes age, history of a mTBI/concussion (excluding moderate, severe, or penetrating TBI), presence of depressive symptoms, and absence of metal, brain lesion, implanted devices, or other exclusionary conditions (see Supplementary Table S1 for more detailed inclusion/exclusion criteria).

### 2.3 Power analysis for primary efficacy outcome

During the initial design phases, the trial was simulated under a variety of alternative scenarios to assess power. For example, if one of the arms had a 4-point improvement over sham, the power was 81–82% with an expected sample size of 150 patients. Alternatively, 95% power and an expected sample size of 137 patients was obtained if one of the arms had a 5-point improvement over sham. If both arms were equal to sham (e.g., the null hypothesis), then the expected trial sample size was 147 patients. This expected sample size is an average of the three possible trial sizes averaged over the probability each sample size occurs [i.e., 99 (if the trial stops after the first interim analysis), 148 (if the trial stops after the second interim analysis), or 198 (if the trial continues to enroll until the maximum number of participants have completed the study)], assuming 10% dropout rate.

The interim analyses allow for efficient stewardship of resources. While there is no guarantee the trial will stop before the maximal sample size, there is substantial probability of meaningful sample size savings based on the outcomes of these scenarios. We also utilize a gatekeeping approach by making the testing of secondary outcomes dependent on the success of the primary analysis.

The Bayesian adaptive approach allows for simultaneous examination of the two arms compared to sham, thus resulting in a more efficient trial. Accumulation of data over the course of the trial is utilized to focus enrollment on the treatment arm that demonstrates superior efficacy. By focusing resources on the better performing arm, we obtain equivalent power as a nonadaptive trial with a high probability of sample size savings, as well as treating participants within the trial better than using equal randomization among all three arms (42).

### 2.4 Procedures

#### 2.4.1 MRI safety assessment

All potential participants undergo a comprehensive MRI safety assessment carried out by trained study personnel. The magnetic resonance imaging (MRI) scan may be contraindicated for participants with ferromagnetic implants, metallic shrapnel, braces, metal piercings, jewelry, or other metallic devices in critical locations in the body. Before scheduling the MRI, participants are asked about embedded metal by the study team (i.e., TMS Screener and Medical History Review). Additionally, the site PI and study team determine participant eligibility during a case conference or similar communication, after reviewing all relevant information. If a contraindication, such as shrapnel, is identified and more information is needed, it may be necessary to schedule an x-ray and consult with site radiologists.

During the MRI visit, the institutional MRI Technologist screens the participant again for the presence of embedded metal using the site’s comprehensive institutional MRI screening form. If the MRI Technologist has any concerns about the safety of the MRI procedures prior to conducting the scan (i.e., positive metal screening), the participant is referred as a patient to the chief of imaging/radiology or other clinical radiologist for follow-up determinations prior to completing the MRI portion of the study. MRI visits may need to be postponed until it has been deemed safe for the completion of the MRI. Participants with non-removable ferromagnetic metal in critical locations in the body are excluded from participating in the study based on the inclusion/exclusion criteria. The study team also maintains a “Positive Metal Screening Tracker” and collects signatures from the determining party.

#### 2.4.2 MRI acquisition

MRI scans are performed at Baseline, Post-aiTBS and the 6-month Follow up evaluation. A 3.0 Tesla MR Scanner equipped with a high-performance gradient subsystem is used at each site (see Supplementary Table S2 for details of MRI acquisition parameters at each site). All participants have a vitamin E capsule placed on their scalp prior to the MRI scan. The vitamin E capsule is placed using the Beam F3 method of identifying the DLPFC described above. This vitamin E capsule is visible in the resulting MRI images and is used to indicate the targeting location for those randomized to the scalp-based targeting and sham arms. To maintain the blind and allow for exploratory analyses comparing stimulation target across arms, all participants have a vitamin E capsule placed on their scalp.

Anatomical imaging includes a high-resolution three-dimensional magnetization prepared rapid acquisition gradient echo (3D-MPRAGE) T1-weighted scan with additional T2-weighted, T2-weighted Fluid Attenuated Inversion Recovery (FLAIR), and susceptibility-weighted imaging sequences. Participants are asked to lie still at rest but are allowed to close their eyes if desired. The anatomical MRI scans last approximately 30 min. The individualized targeting procedure requires a high-resolution T1-weighted anatomical image for alignment to a template space and for cortical surface reconstruction (43).

Resting-state functional MRI uses a dynamic, blood oxygen level dependent MRI sequence. During the scan, participants are asked to lie still at rest, fixate their vision, and let their mind wander. The resting-state functional MRI scan lasts approximately 30 min. These longer scans provide more reliable individualized connectome maps than shorter scans (44).

#### 2.4.3 Targeting

Regardless of targeting strategy, all participants undergo both structural MRI and rsfMRI. All imaging scans are processed centrally by an unblinded study team member. Once the Baseline MRI scans are processed, the optimal coil placement coordinates and coil orientation are returned to the site through a file that can be opened with the site Brainsight neuronavigation system (Rogue Research, Inc.) running version 2.5.4 or later. Through frameless stereotaxy, Brainsight guides TMS operators to the optimal position and orientation of the rTMS coil on the participant’s head to stimulate the targeted brain region. Brainsight neuronavigation system is used for all participants (independent of their randomized arm) to maintain the blind and to maintain consistency of stimulation target across sessions.

For the scalp-based targeting aiTBS arm, we use the Beam F3 method (45), a well-validated approach for localizing prefrontal cortex stimulation in non-invasive brain stimulation studies. All participants randomized to scalp-based targeting receive stimulation over the prefrontal cortex region defined as the location on the scalp that corresponds to the F3 electrode placement given by the International 10–20 system. For the sham aiTBS arm, we use the same Beam F3 targeting strategy as described above with the sham coil. For the rsfMRI targeting, target selection is performed based on rsfMRI data that are pre-processed to remove motion artifacts and then individually parcellated into resting-state networks according to an algorithm detailed in Kong et al. (46), and electric field modeling. Whole-brain connectivity for the Dorsal Attention Network (DAN) and the Default Mode Network (DMN) is computed based on DAN and DMN boundaries identified using the Kong parcellation. The rTMS targets in the rsfMRI-targeted aiTBS group are defined as a cluster in the left dorsolateral prefrontal cortex with largest differences between DAN and DMN connectivity (33). Modeling of the TMS-induced electric field at the targeted site is performed to further optimize TMS coil placement and orientation, as well as correct for scalp-to-target distance differences between the motor cortex and the dorsolateral prefrontal cortex target.

#### 2.4.4 Electric field modeling and optimization

**Figure 2:**
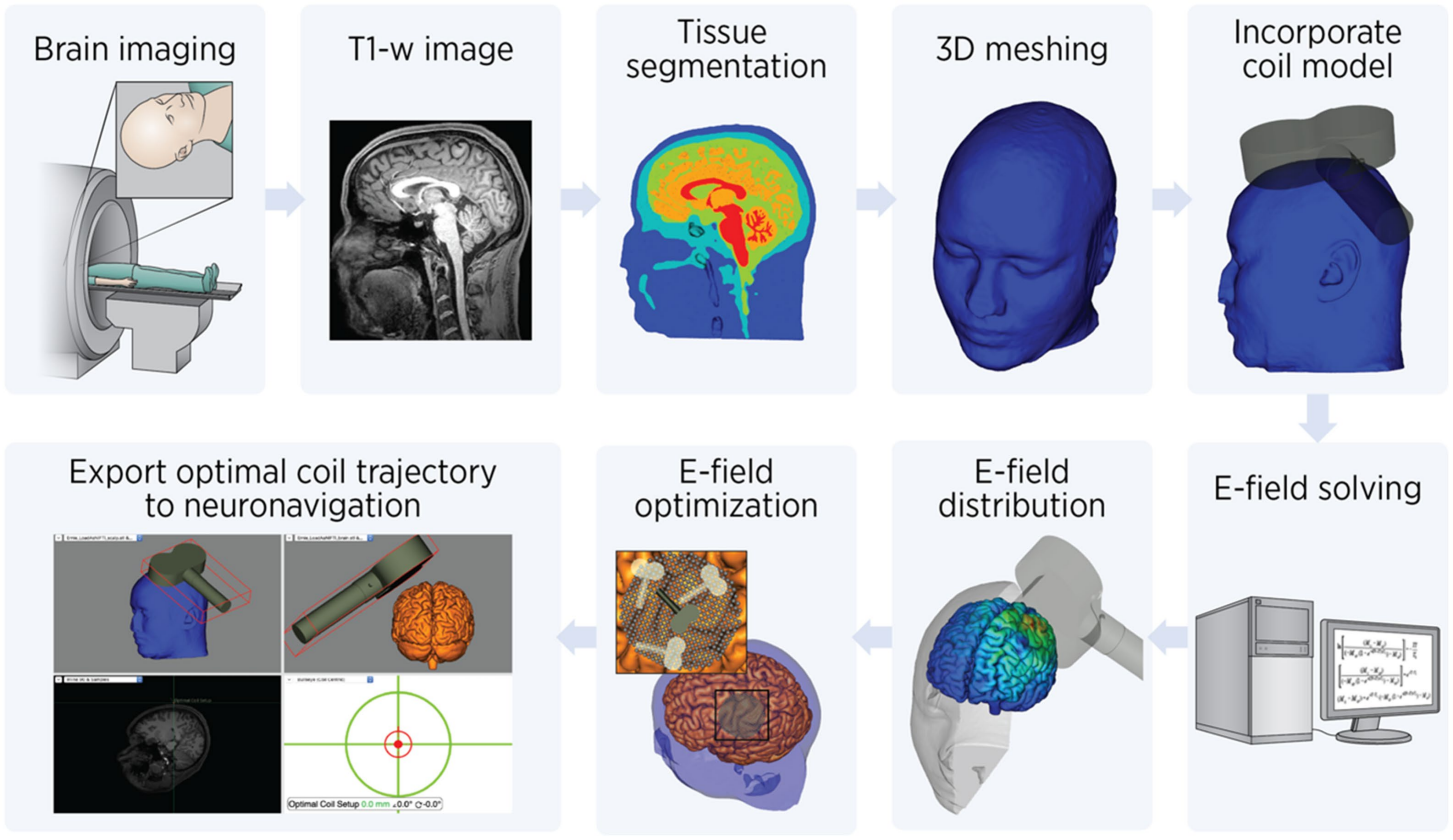
Electric field modeling workflow. Step-by-step workflow for electric field (E-field) modeling TMS: Brain Imaging: The process begins with acquiring *T1-weighted anatomical MRI images* of the subject’s brain. *Tissue segmentation:* The MRI data is segmented into distinct tissue types, such as skin, skull, cerebrospinal fluid (CSF), gray matter, and white matter. This step is crucial for assigning tissue-specific properties in the model. *3D meshing*: A computational head model is created by converting the segmented tissues into a mesh composed of small tetrahedral elements. This mesh allows for finite element analysis to simulate the E-field. *Incorporate coil model*: The TMS coil model is integrated into the head mesh. Its position, orientation relative to the head, and pulse intensity are specified to simulate the E-field generated during stimulation. *E-field solving*: Using finite element methods (FEM), Maxwell’s equations are solved to calculate the E-field distribution within the head. *E-field distribution*: The resulting E-field distribution is visualized and quantified to evaluate the spatial targeting of the stimulation. *E-field optimization*: Coil placement and orientation are optimized to maximize the E-field intensity at the target brain region. This involves testing multiple positions and angles to identify the best configuration. *Export optimal coil trajectory*: Finally, the optimized coil position and orientation are exported to a neuronavigation system. This ensures precise replication of the simulated coil trajectory during the actual TMS procedure.

The process of E-field modeling for TMS involves multiple steps, each designed to ensure accurate simulation and optimization of brain stimulation. This workflow is illustrated in Figure 2, which breaks down the process into clear, sequential stages. The study team acquires T1-weighted anatomical MRI scans, providing detailed structural information about the subject’s head and brain. These images serve as the foundation for creating a subject-specific computational model. In the preprocessing step, the vitamin E fiducial marker visible on the MRI images is manually removed using ITK-SNAP (47) to avoid interference with segmentation and modeling. Subsequently, head model construction, meshing, and electric field computation are performed using SimNIBS (48). First, the cleaned T1-weighted image undergoes tissue segmentation to classify different tissue types such as skin, skull, cerebrospinal fluid (CSF), gray matter, and white matter. This segmentation process is essential for modeling the unique electrical properties of each tissue. Following segmentation, the data is used to generate a 3D computational mesh. This mesh represents the head as a collection of small tetrahedral elements with an assigned tissue-specific electrical conductivity value. Isotropic conductivity values are assigned to the elements based on tissue type: skin 0.465 S/m, skull 0.010 S/m, cerebrospinal fluid 1.654 S/m, gray matter 0.265 S/m, and white matter 0.126 S/m.

Once the head model is prepared, the TMS coil model is incorporated into the simulation (Figure 2, rightmost, top row). The coil’s position and orientation relative to the head is specified to calculate the E-field generated during stimulation. The calculations use finite element methods (FEM) to solve Maxwell’s equations, enabling precise estimation of the E-field distribution within the head. The E-field distribution is visualized and quantified to identify areas of high E-field intensity in relation to the brain’s anatomy. This informs whether the stimulation is effectively targeting the desired brain region. Next, E-field optimization is performed. Using the TAP software (49), a systematic search is conducted to determine the optimal coil placement and orientation, leveraging advanced computational methods. This involves evaluating coil positions on a fine 1-mm grid and testing orientations in 4-degree increments. The goal is to maximize the E-field intensity within a 20-mm radius around the target brain coordinates.

As stimulation is delivered at 90% of resting motor threshold at the cortical target (DLPFC) and corrected for scalp-to-target distance, the difference between scalp-to-target distance at DLPFC versus the primary motor cortex (where resting motor threshold is obtained) needs to be calculated. Thus, the model calibrates stimulation intensity by comparing the E-field strength between M1 and the target region, accounting for scalp-to-cortex distance variations. This calibration uses the motor threshold—the smallest amount of stimulation needed to produce a visible muscle response—as a baseline for scaling. Finally, the optimal coil trajectory is exported to a neuronavigation system to ensure precise replication of the simulation during treatment. This step ensures that the optimized coil position and orientation from the simulations can be precisely replicated during the actual TMS treatment, enhancing targeting accuracy and reproducibility. The coil orientation and target parameters are saved in a text file and imported into the Brainsight neuronavigation system (version 2.5.4, Rogue Research, Canada). The Brainsight system employs frameless stereotaxy to guide TMS operators to the precise stimulation target on the participant’s scalp, ensuring accurate and consistent delivery across sessions while preserving blinding between study arms.

#### 2.4.5 Sham blinding

The Magstim Double 70 mm Air Film sham coil has a figure-of-eight winding with components that largely cancel the field produced under the center of the coil. The sham coil is designed to simulate the sounds and vibrations of the active coil while inducing superficial currents using a brief pulse of electromagnetic energy that is not sufficient to stimulate the cortex. The Magstim Double 70 mm Air Film sham coil additionally mimics the cutaneous sensations (e.g., tingling, muscle twitching) that are produced by the active version of the coil. Previous validation testing on earlier models of the Magstim sham coils using TMS-naïve volunteers indicate validity with 12 of 15 participants in the sham group and 11 of 13 participants in the active group believing they received active stimulation (50). We therefore believe that this coil has equal or better blinding than previous models. Nonetheless, we plan to enroll only TMS-naïve participants to preserve blinding since participants who have experienced active TMS may be able to distinguish active from sham TMS more easily.

To further protect the blind, the study team has a strict data collection and management plan. Site staff only have access to data and collection forms that are required for their role. We collect information about the validity of the blind from both the participant and TMS operator, after the first TMS session and at completion of the final TMS session, in two separate self-report measures. The self-report blinding assessments are inaccessible to blinded staff so as not to bias their outcome scoring. Role permissions and randomization in the electronic data capture system are handled and maintained separately by an unblinded data management team.

#### 2.4.6 Resting motor threshold

Prior to the first rTMS session, resting motor thresholds are assessed by a privileged provider at each site by administering several single pulses of TMS over the left (dominant) motor cortex. The locations of the appropriate part of the motor cortex are initially estimated as approximately 5 cm lateral to the vertex of the head, and the location/intensity of stimulation is titrated using a parameter estimation by sequential testing (PEST) algorithm (51) until a contraction is visually observed in the contralateral abductor pollicis brevis or the first digital interosseous muscle 50% of the time.

#### 2.4.7 Intervention: aiTBS sessions

**Figure 3:**
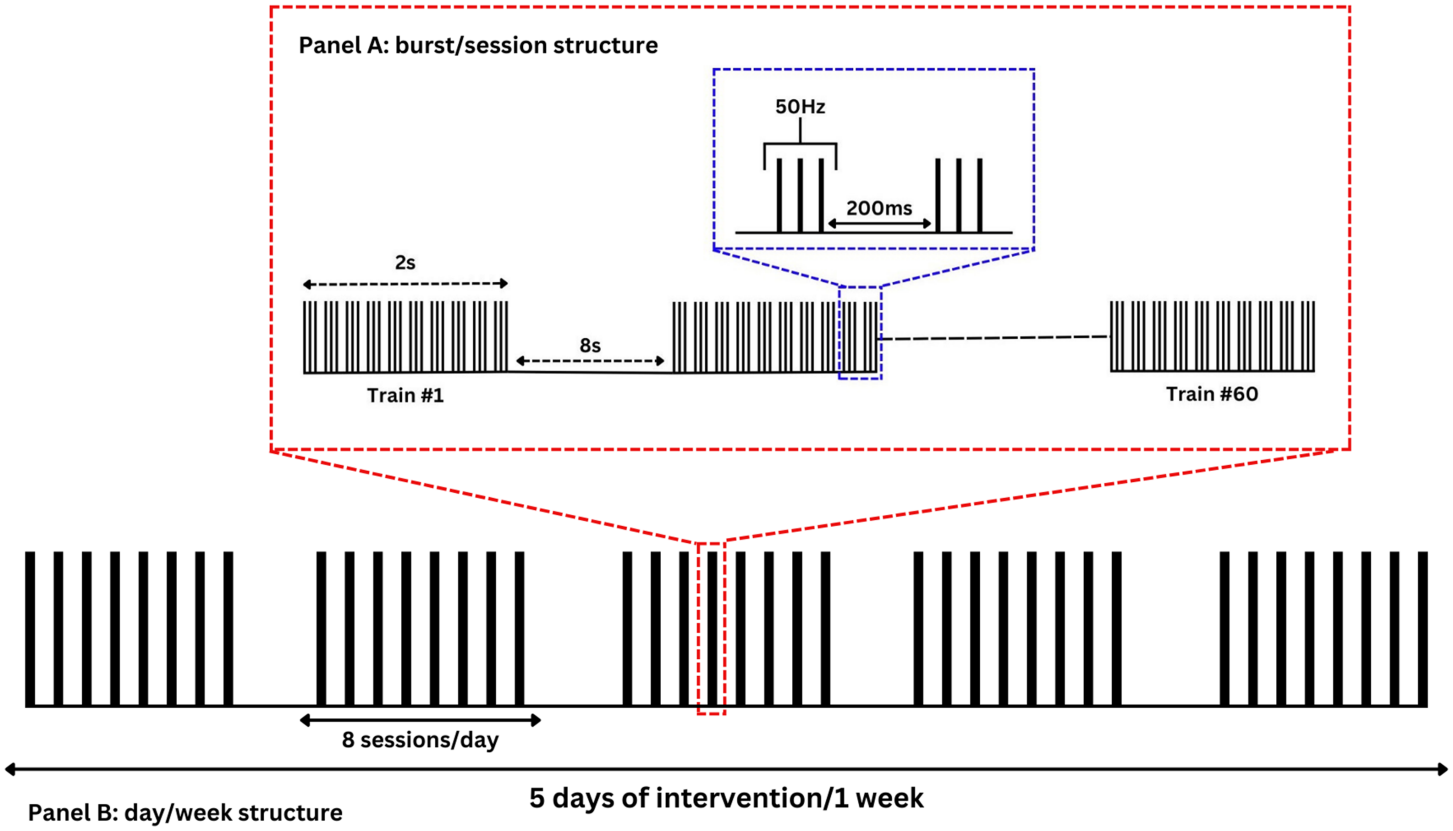
Accelerated intermittent theta burst stimulation. **(A)** iTBS sessions consist of 60, 2-s, trains of 10 bursts. Bursts consist of three rTMS pulses at 50 Hz applied every 200 ms (5 Hz). An 8-s interval separates each train; the total time it takes to complete 60 trains in a session is approximately 9 min and 30 s. **(B)** The intervention phase consists of 40 iTBS sessions provided over a consecutive 5 business day period with 8 sessions provided per day. The 5-day period may take place over a 7 calendar-day week.

Figure 3 illustrates the intervention phase of the study, which consists of 40 iTBS sessions provided over a 1-week (five business days) period (eight sessions/day Monday–Friday). Eight sessions/day are used instead of the 10 sessions/day used in the SNT protocol because of logistical and staffing constraints at the research sites. Each iTBS session consists of 60, 2-s, trains of 10 bursts. Bursts are applied every 200 ms (5 Hz). Each burst consists of three pulses of rTMS at 50 Hz. There is an 8-s intertrain interval such that each session takes approximately 9 min and 30 s in total. Stimulation sessions are delivered every 60 min (50-min break from the end of one session to the beginning of another). Stimulation is delivered at 90% of resting motor threshold at the cortical target and corrected for scalp-to-target distance (48). If the participant cannot tolerate 90% of resting motor threshold at the first session, a titration schedule is followed such that they are allowed to receive as low as 70% of resting motor threshold on the first and second sessions, and 80% on the third session. All participants must receive iTBS at 90% of resting motor threshold by the fourth session and remain at this intensity for the rest of the treatment protocol.

Primary analyses will be conducted with an intention to treat manner with all participants who receive at least one session of aiTBS. A secondary analysis will be done per protocol including participants who complete at least 32 of the 40 sessions (80%).

#### 2.4.8 Baseline, post aiTBS, and follow-up behavioral evaluations

Participants complete several self-report questionnaires, structured interviews, and MRI scans both for determination of eligibility as well as baseline symptom characterization, aiTBS targeting, and functional network mapping. The Post-aiTBS and follow-up evaluations consist of repeating assessments and MRI scans that were conducted at the Baseline Evaluation (see Supplementary Table S3 for a list of measures that are administered over the course of the study). The Post-aiTBS Evaluation is conducted within 10 working days of the participants’ final rTMS session. Participants then complete regular follow-up evaluations for up to a total of six-monthly sessions.

Participants are afforded the opportunity to opt in or out of individual follow-up evaluations. The research team make up to four attempts to contact the participant via phone/email. If the participant fails to respond to the study team’s attempts at contact, it is considered an unwillingness to participate during the specified month. Contact with the participant is reinitiated for the subsequent follow-up evaluations. If they choose not to participate in follow-up evaluations, their data may not be included in secondary outcome analyses related to follow-up time points. During the Follow-up phase of this study, participants are allowed to change their treatment regimen including the addition of new or modified interventions (psychopharmacological, behavioral, or device-based treatments). The study team monitor these changes and they are also integrated into the statistical model as co-variates for the secondary outcome analyses related to follow-up time points.

To ensure robust administration and scoring of the study’s primary outcome measure, the study team has developed a comprehensive training plan to standardize MADRS administration across performance sites. The MADRS administration training plan utilizes a 4-phase format that required (1) didactic reading, (2) observations, (3) practice sessions, and (4) certification by a trained investigator. Trainers utilize a Rater Applied Performance Scale (RAPS) (52), which has been applied to neuropsychological assessment training, to objectively rate the administrator’s performance; a score of 3 or higher on a 4-point Likert scale indicates a “good” performance and subsequent certification. In general, most MADRS administrations for each individual participant are performed by the same team member.

#### 2.4.9 Optional maintenance aiTBS sessions

Participants who complete at least 32 randomized aiTBS sessions, achieve treatment response (≥50% improvement in MADRS), and experience a remission of symptoms (MADRS ≤ 10) at the Post-aiTBS evaluation, followed by a relapse of depressive symptoms (defined as MADRS ≥ 15) during the course of the six-month Follow-up period will be offered optional Maintenance aiTBS sessions. Regardless of randomized group assignment (i.e., active arms vs. sham arm), optional Maintenance aiTBS sessions will be offered to all participants who meet the specified criteria. Participants will be able to take part in Maintenance sessions regardless of original group assignment (i.e., the study will remain double-blinded). If the participant meets the above criteria and expresses interest in receiving the optional Maintenance aiTBS sessions, the research team will perform an additional screening process to confirm continued eligibility including completion of a screening form as well as conducting a review of relevant medical records inclusive of records that have been entered into the EMR system since the initial baseline review.

The Maintenance course of aiTBS will be identical to the initial randomized phase of the study where the participant will receive up to 40 sessions of aiTBS across 5 business days (Monday–Friday). Resting motor threshold will be re-assessed since it may change over a several month period. Participants will receive the same protocol they received during the Randomization phase during this optional Maintenance phase and will remain blinded. Participants who decline or do not complete the Maintenance sessions may continue to take part in the Follow-up Phase of the study. Participants will only be able to partake in a single Maintenance course during the six-month follow-up period and will not be able to partake in any additional Maintenance courses of aiTBS as part of the research study. Maintenance treatments or other major changes to their clinical regimen will be considered as co-variates in secondary analyses of outcomes obtained during the Follow-up Phase of the study.

### 2.5 Data analysis

#### 2.5.1 Primary analysis of primary efficacy outcome

We propose a Bayesian adaptive trial with interim analyses allowing for dropping of inferior active arms as well as futility and success stopping. The trial will begin by enrolling all three arms (sham and 2 active arms) in a 1:1:1 allocation with non-informative prior probabilities that any arm is superior to any other arm. Interim analyses will be conducted when *N* = 90 and *N* = 135 have been enrolled. At each interim analysis the following adaptations may be made

1. Arm dropping – If either active arm has a 60% or greater Bayesian posterior probability of being superior to the other active arm, the inferior active arm will be dropped from the study, with the remaining trial maintaining the 2:1 active:sham randomization, but with only one active arm.
2. Futility stopping – If neither active arm has greater than a 60% Bayesian posterior probability of being superior to sham (this will occur only if both arms are performing only slightly better than sham or worse), then the entire trial will stop for futility. This will provide proper stewardship of resources by not continuing a trial that is unlikely to yield a successful conclusion.
3. Success stopping – If either active arm obtains a 99.75% posterior probability of being superior to sham, the trial will stop for success. If this high bar is met early, the trial will allow early dissemination of this successful result. Reaching this high bar will require very convincing data at an interim analysis.

If the trial continues to the maximal sample size, then the trial will be deemed successful if either active arm achieves a 99% posterior probability of superiority to sham. The 99% threshold was determined through simulation (see FDA guidance on adaptive trials). When combined with the early stopping rules, the full trial will maintain 2.5% type 1 error accounting for both the multiplicity of 2 arms and the 2 interim analyses, based on 100,000 simulations.

#### 2.5.2 Analysis of primary safety outcome

For adverse events (AEs) with low incidence rates, resulting in sample sizes below that required for formal statistical treatment, descriptive statistics will be utilized. For events with rates above 10%, where formal statistical tests are possible, we will utilize chi-squared tests between the sham and pooled active arms to test for an increased risk in the active arms.

#### 2.5.3 Secondary analyses

This study is powered to detect significant effects in the primary analysis (described above) of the primary efficacy outcome (MADRS). In addition to this primary analysis, we propose to conduct secondary analyses. If the primary analysis of the primary outcome measure is successful, all secondary outcome measures will be used to assess the robustness of the results. If the primary analysis of the primary outcome measure is not successful, all secondary outcome measures will be considered exploratory/hypothesis generating. We will apply the Benjamini-Hochberg procedure to control for the false discovery rate when testing multiple secondary outcomes (53). Planned analyses, hypotheses, and statistical tests are shown in Supplementary Table S4.

#### 2.5.4 Exploratory analyses

Simulations will evaluate differences in MADRS scores as well as secondary outcome measures among treatment arms, co-varying for baseline characteristics that could identify responders vs. non-responders to rTMS (i.e., demographic characteristics, baseline depressive symptom severity, type or number of TBIs, Combat Exposure, Performance on the NIH Toolbox or TOMM, PTSD, repetitive negative thinking (RNT), substance-use, treatment-resistance, duration of depression, MRI characteristics). If subgroups or co-variates can be identified, their characteristics may be used as inclusion/exclusion criteria in future clinical trials.

An ANOVA will be used to compare the distribution of targeted regions within the DLPFC between the two targeting strategies. An ANOVA will also be conducted to evaluate whether there is a difference in the number of participants who participate in behavioral therapy during the follow-up period across the treatment arms. Mediation analyses will also be conducted to evaluate whether participation in behavioral therapy mediates the relationship between type of rTMS (active or sham) and clinical outcomes during the follow-up period.

## 3 Discussion

This novel non-invasive intervention has the potential to provide a rapid and effective treatment for SMs and veterans with depressive symptoms and a history of concussion, filling the gap for many who have not responded to conventional treatments. To our knowledge, the current study is the first to assess the efficacy and safety of rsfMRI-targeted aiTBS in SMs and veterans with a history of concussion. This study will also provide evidence of whether individualized neuronavigation adds value relative to less resource-intensive targeting strategies in this clinically complex population. The randomized, sham-controlled approach with both clinical and imaging outcomes can provide data toward explanatory neural mechanisms for aiTBS treatment efficacy and potentially identify biomarkers of treatment response.

The study uses three different MRI scanners (Siemens Magnetom Skyra, Siemens Magnetom Prisma, and GE Signa Premier) across the three different performance sites. To test reliability within and across these scanners, prior to initiating the study, we performed multiple test scans on healthy volunteers at each of the sites. We assessed image quality and test–retest reliability of the rsfMRI-based network mapping and TMS target identification on the test scans. The network mapping and TMS target identification yielded very similar results at each of the sites, with excellent test–retest reliability. These results provided confidence that the three different MRI scanners used at the three sites would provide similar quality TMS targeting. From a clinical applicability and generalizability perspective, the heterogeneity of MRI scanners should be considered a strength of the study design. If the rsfMRI-guided TMS treatment will be used more broadly, it will need to be applicable in a generalized multicenter fashion using a variety of MRI scanners.

This study is not without limitations. The first is the inherent patient burden of any rTMS intervention as compared to pharmacotherapy. rTMS requires the patient to come to a clinic and the clinic to be resourced with the appropriate devices and trained/credentialed staff to administer the treatment. Compared to standard (once per day for 6 + weeks) rTMS treatment regimens, the full aiTBS treatment course can be administered in 5 days. Still, patients may need to take medical leave or be approved by command for alternate duty location for the one-week treatment. Medical leave is usually feasible with sufficient planning. The treatment sessions are applied for 10 min out of each hour. Thus, depending on the flexibility of the participant’s work situation, telework or other activities may be feasible in an adjacent private office space near the treatment facility during the 50-min periods between aiTBS sessions. It could also be appropriate for maintenance therapy in individuals who have responded to aiTBS in the past and may be showing early signs of relapse.

This study is powered based on the primary outcome measure (MADRS), however, given the known heterogeneity in response to rTMS, we are collecting several additional, secondary and exploratory outcomes including measures of PTSD (PCL-5), executive functioning (NIH Toolbox), quality of life (TBI-QOL), and demographic information (e.g., treatment resistance and combat experience). That being said, these measures add burden and time to the study procedures and are not all inclusive of all potential moderators/mediators of response. Though it would be infeasible to power the study for these variables and the combination of them, they will be considered in the secondary and exploratory analyses, which will provide preliminary data and inform future studies specifically exploring their role in response to treatment. As the study is not powered to make strong claims about these endpoints, we will be careful to not overinterpret the outcomes of these analyses and utilize strategies to control for the false discovery rate. Additionally, we expect to enroll fewer women than men in the current study sample due to the demographics of the military population. Treatment outcomes and optimal treatment parameters may differ between the sexes, but the current study will likely not be powered to assess those differences. Future studies will be important to evaluate these sex differences.

The design of this study and data obtained may also provide information relevant to the understanding of the pathophysiological mechanisms underlying symptoms related to other military relevant conditions including repeated subconcussive blast exposures, suicidal behaviors, anomalous health incidents, long COVID, PTSD, suboptimal cognitive performance, and other brain health-related conditions. For such indications we would start with observational studies involving high quality resting state fMRI-based individualized connectome mapping to learn about the brain networks affected. From there, we could determine whether there are rational stimulation sites that could be targeted by rsfMRI-targeted aiTBS. The stimulation sites for other brain health-related conditions could be the same as in depressive symptoms associated with concussion or differ in important ways. For example, aiTBS specifically targeting suicidality may differ in number of sessions or frequency of sessions as compared to broad depressive symptoms. This is a potentially very fruitful line of research for additional studies; we are committed to sharing resources and best practices with other investigators working in these domains.

## 4 Ethics and dissemination

If proven effective, aiTBS could be implemented in an inpatient or outpatient clinical care setting. It could be administered either as a stand-alone or adjunctive treatment in the context of a multidisciplinary program combined with other activities such as cognitive behavioral therapy, art therapy, music therapy, speech therapy, occupational therapy, physical therapy, family education, etc. In clinical practice, as we expand the number of people receiving aiTBS, we will learn about its interactions with other therapies. Future research studies will be required to assess the effects of aiTBS paired with emerging treatments such as ketamine, ibogaine, virtual reality-enhanced cognitive behavioral therapy, and others. While not directly studied in this trial, it is reasonably likely that older individuals, children/adolescents, and pregnant women may also benefit. aiTBS could be performed in a specialized clinic with trained medical providers, staff, and specialized equipment. Multidisciplinary outpatient programs, such as those at the Intrepid Spirit Centers serving active-duty SMs and VA Polytrauma System of Care facilities, currently serve patients with TBI and concomitant depression on a regular basis around the country. The VA has also recently rolled out a large-scale clinical TMS program at several of their medical centers in Psychiatry departments.

The rsfMRI targeting likely would only be available at a role 4 (long-term care) medical facility with sophisticated MRI technology. Scalp-targeted aiTBS, however, could be performed in a role 3 (comprehensive care) facility if it proves to be effective. Treatment in a role 3 facility could potentially prevent a deployed service member from requiring full evacuation from theater. The required TMS equipment uses standard electrical power and is easy to transport.

We estimate that the cost of ICT-aiTBS in clinical practice would be approximately $9,500 per course of treatment. This is comparable to the costs of inpatient hospitalization and to electroconvulsive therapy, but substantially less than other treatments such as multi-week multidisciplinary intensive outpatient programs. The cost in terms of lost productivity of a person suffering from inadequately treated severe depression can be substantial. Economic analyses indicate that even standard rTMS protocols are cost effective, at an estimated $35,000 per quality adjusted life year gained (54). ICT-aiTBS may be substantially more effective than other forms of TMS, and so the cost effectiveness may be even greater.

aiTBS has the potential to fundamentally change our current treatment algorithm for treatment-resistant depression following concussion and provide hope to those who have been long suffering with limited treatment options. Current or former SMs who have sustained one or more concussions have significantly higher rates of depressive symptoms compared to non-injured peers. Suicide is the number one TBI related cause of death in this cohort. The severity of depression following concussion is strongly correlated with global disability, rate of recovery, and quality of life (QOL). Untreated depressive symptoms in SMs may lead to early discharge from service and decreased medical/personnel readiness of the military force. Thus, improvements in existing treatments and innovative novel interventions are critically needed to improve the mental health outcomes of SMs and veterans. A long-lasting and effective treatment that provides relief from depressive symptoms in this context would be expected to improve global disability and rate of recovery, save lives by reducing suicidality, and improve military resiliency/fighting strength, service member retention, and QOL. Further, aiTBS could reduce health care cost by reducing overall utilization of mental health hospitalizations and rehabilitation services. If successful, in the near-term this project will accelerate solutions to improve the health, well-being, and healthcare of SMs and veterans with depressive symptoms following concussion.

## Author disclaimer

SL is an inventor on a patent on TMS coil design (no royalties). The opinions expressed in this article are the authors’ own and do not reflect the views of the National Institutes of Health, the Department of Health and Human Services, or the United States government. Z-DD is an inventor on patents and patent applications on electromagnetic brain stimulation technologies and methods of dosing held by the National Institutes of Health, Columbia University, University of New Mexico, and NEVA Electromagnetics (no royalties).
