# Supplementary material for: Adaptive trial for the treatment of depressive symptoms associated with concussion using accelerated intermittent theta burst stimulation (ADEPT): rationale, design and methods
Source: Front Neurol. 2025 Jun 13;16:1605157. doi: 10.3389/fneur.2025.1605157 (PMC12202228; doi:10.3389/fneur.2025.1605157)
Supplement: Supplementary file 1 [file Table_1.docx]

**Supplementary Table 1. Inclusion/Exclusion Criteria**

| Inclusion Criteria | Exclusion Criteria |
| --- | --- |
| 1. Age:18-55 years old 2. Current or former US military service member eligible for care at a MTF or VAMC 3. Self-reported or medically diagnosed history of concussion >6 months, but <26 years prior to consent, as defined below    1. Positive Loss of Consciousness of <30 minutes    2. Positive Alteration of Consciousness of <24 hours    3. Positive Post-traumatic Amnesia of 0 to 1 day 4. Baseline MADRS >13 at the time of screening 5. Maintained a steady psychotropic medication regimen for six weeks and a steady behavioral therapy regimen for twelve weeks prior to enrollment in the study    1. Participants will be urged to maintain their medication and behavioral therapy regimen throughout the study, unless otherwise recommended by the personal physician.    2. Participants who are not on a psychotropic medication regimen or behavioral therapy regimen will be urged to refrain from starting new treatment until after the study    3. Changes in medication and/or behavioral therapy regimens will be reviewed and assessed with the study team before participants can continue their enrollment 6. Participants of childbearing potential must agree to use an effective method of birth control for the duration of the trial.    1. Least effective methods include male condom, female condom, cervical cap, fertility calendar, and spermicide    2. Most effective methods include implant, IUD, sterilization, injection, pill, patch, and vaginal ring 7. Participants must be under the care of a primary care physician and/or behavioral health provider. | 1. Elevated risk of seizures at the time of rTMS including any of the following:    1. History of unprovoked seizures or any seizure within 24 hours of sustaining a concussion(s) or other head injury regardless of whether it was determined to have been related to concussion.    2. Family history of two or more unprovoked seizures in a first degree biological relative    3. History of Moderate or Severe TBI    4. History of Penetrating TBI    5. Intracranial lesion that would increase seizure risk.    6. Currently taking medication or other substances that substantially lowers the seizure threshold 2. Contra-indications to an awake 3T MRI without contrast at the time of the Baseline MRI 3. Severe claustrophobia interfering with medication/sedation-free 3T closed-bore MRI 4. Intracranial lesion that would produce an artifact that would compromise the integrity of rsfMRI data 5. History of severe or recent uncontrolled heart disease 6. Presence of a cardiac pacemaker or intracardiac lines 7. Any implant, prosthesis or other permanent alteration of the body that would be unsafe with MRI or TMS or that would produce an artifact that would compromise the integrity of data 8. Presence of rapidly progressive illnesses 9. History of Bipolar Disorder or Schizophrenia Spectrum Disorders 10. Current evidence of substance-induced mood disorder, active psychosis, or depression secondary to general medical illness other than TBI 11. Concomitant or lifetime history of receiving open-label TMS, due to issues preserving blinding. 12. Concomitant or recent history (within 6 months prior to enrollment) of receiving other neurostimulatory treatment (including but not limited to transcranial direct current (tDCS), transcranial alternating current (tACS), alpha stim, or electroconvulsive therapy) 13. Suicide attempt within six months prior to enrollment 14. Right upper extremity amputation or other condition precluding left motor threshold calibration 15. Inability to complete timeline of study 16. Any considerations that, in the opinion of the investigator, may adversely affect participant safety, participation, or the scientific validity of the data being collected |
