# Supplementary material for: Adaptive trial for the treatment of depressive symptoms associated with concussion using accelerated intermittent theta burst stimulation (ADEPT): rationale, design and methods
Source: Front Neurol. 2025 Jun 13;16:1605157. doi: 10.3389/fneur.2025.1605157 (PMC12202228; doi:10.3389/fneur.2025.1605157)
Supplement: Supplementary file 2 [file Table_2.docx]

**Supplementary Table 2. MRI Parameters for each Site**

| Site | Scanner | Head Coil | Gradient Parameters | rsfMRI Scan parameters | MPRAGE Parameters | BRAVO Sequence Parameters |
| --- | --- | --- | --- | --- | --- | --- |
| William Beaumont Army Medical Center | Siemens Magnetom Skyra | 32-channel | 45 mT/m @ 200 T/m/s | TR=2610ms,  TE=20 ms;  flip angle=60⁰,  voxel size=3mm isotropic,  582 time points | TR=2300ms,  TE=3.46ms,  flip angle=12⁰,  voxel size=0.469x0.469x1.2mm | N/A |
| George Mason University  (ATAMMC Outsource) | Siemens Magnetom Prisma | 32-channel | 80 mT/m @ 200 T/m/s | TR=1500ms,  TE=33 ms,  flip angle=45⁰,  voxel size= 3mm isotropic,  1035 time points | TR=2400ms,  TE=2.28ms,  FA=8⁰,  voxel size =0.8mm isotropic | N/A |
| VA Palo Alto | GE Signa Premier | 21-channel | 80 mT @ 200 T/m/s | TR=2200ms,  TE=17.5 ms,  flip angle=60⁰,  voxel size =2.25x2.25x3mm, 750 time points | N/A | TR=7.29ms,  TR=3ms,  FA=12⁰,  voxel size= 0.469x0.469x1.2mm |
