# Supplementary material for: Adaptive trial for the treatment of depressive symptoms associated with concussion using accelerated intermittent theta burst stimulation (ADEPT): rationale, design and methods
Source: Front Neurol. 2025 Jun 13;16:1605157. doi: 10.3389/fneur.2025.1605157 (PMC12202228; doi:10.3389/fneur.2025.1605157)
Supplement: Supplementary file 3 [file Table_3.docx]

**Supplementary Table 3. Measures Administered over the Course of the Study**

| Name | Purpose | Frequency | Description |
| --- | --- | --- | --- |
| Demographics Questionnaire | Enrollment | Baseline | The Demographics Questionnaire contains questions pertinent to this research effort, including age, race, ethnicity, biological sex, primary language, handedness, marital status, educational attainment, active military status, current duty status, number of years in military, branch of service, military rank, occupation code/rate, Medical Evaluation Board or Physical Evaluation Board status. The self-report form will be labeled with a study ID, site, study visit time point, and/or date of evaluation and takes approximately 5-10 minutes to complete. |
| Combat Exposure Scale (CES) | Enrollment | Baseline | The Combat Exposure Scale (CES) is a 7-item self-report measure, commonly used in the DoD and VA system to assess wartime stressors experienced by combatants. Respondents are asked to reply based on their exposure to various combat situations, such as firing rounds at the enemy and being on dangerous duty. Items are rated on a 5-point frequency (1 = "no" or "never" to 5 = "26+ times" or "51+ times"), 5-point duration (1 = "never" to 5 = "7+ months"), or 45-point degree of loss (1 = "none" to 45 = "76% or more") scale. The CES will take approximately 5 minutes to complete. |
| TBI History Form | Eligibility | Baseline | The TBI History form is designed to elicit information regarding the participant’s TBI(s). The form utilizes the Ohio State University Traumatic Brain Injury Identification Method (OSU TBI-ID)(1) as its standardized procedure for obtaining the lifetime history of an individual's TBI via self-report. This will be administered by trained study personnel and will take approximately 30-45 minutes to complete. |
| Medical History Review | Eligibility | Baseline | The Medical History Review will involve both a review of available medical records as well as a brief (15-20 minute) discussion with the site PI or a qualified designee. The review will collect medical information including personal and family medical history (including current and previous diagnosed conditions), concomitant and previously prescribed medications/therapies, injury-related outcomes, and/or previous neuroimaging results. Participants with personal copies of their medical records can voluntarily provide such records to study personnel as a supplement to electronic medical records. Specific information regarding the previous treatments for depression, treatment resistance, duration of depression, and onset of depression relative to the concussive incident(s) will be recorded and used as potential covariates in the analysis of TMS effects. |
| Concomitant Medications Form | Eligibility | Baseline | The Concomitant Medications form will capture medication, and supplements being taken by the participant. A trained study team member will review and do medication reconciliation using concomitant medications form. The Concomitant Medication form will take 3-5 minutes to complete. |
| Concomitant Therapies Form | Eligibility | Baseline | The Concomitant Therapies form will capture ongoing behavioral health therapies. The form will be administered by a trained study team member who will review concomitant behavioral therapies at each study visit. The Concomitant Therapies form will take 3-5 minutes to complete. |
| Alcohol and Drug Use Screener | Eligibility | Baseline | This will be a 7-item self-report questionnaire, modeled after the Alcohol Use Disorders Identification Test-Concise (AUDIT-C) (2) and Drug Use Disorders Identification Test-Concise (DUDIT-C) (3). This measure consists of questions probing frequency and quantity of alcohol use as well as use of drugs known to reduce seizure threshold. The Alcohol and Drug Use Screener will take approximately 5 minutes to complete. |
| Montgomery-Asberg Depression Rating Scale (MADRS) | Primary Outcome Measure | Baseline, Post aiTBS, and Follow-up | The MADRS is an interview-based rating scale for signs and symptoms associated with MDD (4). The MADRS takes approximately 15-20 minutes to complete and will be administered by trained study personnel. |
| Symptoms of Major Depressive Disorder Scale (SMDDS) | Secondary Outcome Measure | Baseline, Post aiTBS, and Follow-up | The SMDDS is a 16-item self-report measure assessing nine symptom domains of Major Depressive Disorder: 1) negative emotions/mood; 2) anxiety; 3) low energy; 4) cognition; 5) sleep disturbances; 6) self-harm/suicide; 7) low motivation; 8) sense of self; and 9) eating behavior. Respondents rate each item on a rating scale of 0 (“not at all”/“never”) to 4 (“extremely”/“always”). The self-report form takes approximately 5-10 minutes to complete (4). |
| Inventory of Depressive Symptomatology – Self-report (IDS-SR) | Secondary Outcome Measure | Baseline, Post aiTBS, and Follow-up | The IDS-SR is a 30-item patient reported inventory used to assess the severity of the nine diagnostic symptom criteria used in the DSM 1) sad mood; 2) reduced concentration; 3) self-criticism; 4) suicidal ideation; 5) loss of interest and pleasure; 6) energy/fatigue; 7) sleep disturbance (initial, middle, and late insomnia or hypersomnia); 8) decrease or increase in appetite or weight; and 9) psychomotor agitation or retardation, as well as commonly associated symptoms (e.g., anxiety and irritability) and items relevant to melancholic, or atypical symptom features (5). The total score ranges from 0 to 84. The IDS-SR form takes approximately 25-30 minutes to complete. |
| PTSD Checklist for DSM-5 (PCL-5) | Secondary Outcome Measure | Baseline, Post aiTBS, and Follow-up | The PCL-5 is a 20-item self-report measure assessing the DSM-5 symptom criteria for PTSD. Respondents use a self-report rating scale from 0 (“not at all”) to 4 (“extremely”) for each symptom (6). The self-report form takes approximately 5-10 minutes to complete. |
| Perseverative Thinking Questionnaire (PTQ) | Secondary Outcome Measure | Baseline, Post aiTBS, and Follow-up | The PTQ is a 15-item questionnaire developed to assess repetitive negative thinking. Respondents use a self report rating scale from 0 (“never”) to 4 (“almost always”) to assess the characteristics of RNT: (1) repetitive, intrusive, and difficult to  disengage from; (2) unproductive; and (3) capturing mental capacity (7). This self-report will take approximately 5 minutes to complete. |
| TBI Quality of Life Scale (TBI-QOL) | Secondary Outcome Measure | Baseline, Post aiTBS, and Follow-up | The TBI-QOL is a self-report measure assessing areas of physical (e.g., fatigue, headache), cognitive (e.g., executive function), emotional (e.g., anxiety, depressive symptoms), and social (e.g., social satisfaction) functioning specific to those sustaining a traumatic brain injury (8). Each module contains between 6 and 10 items and provides a subscale score for that domain. The measure will take approximately 25-30 minutes to complete. |
| NIH Toolbox for the Assessment of Neurological and Behavioral Function | Secondary Outcome Measure | Baseline, Post aiTBS, and Follow-up | The NIH Toolbox includes a brief, diverse, accessible, and psychometrically sound tablet-based battery of cognitive tests. The tests have well-established norms based on age, sex, and educational attainment (9). It is considered a standard in cognitive performance testing measures. NIH Toolbox modules will be administered by trained study personnel using dedicated NIH Toolbox iPads. The following modules will be used for this study: (1) Flanker Inhibitory Control and Attention; (2) List Sorting Working Memory; (3) Dimensional Change Card Sort; (4) Pattern Comparison Processing Speed; (5) Picture Sequence Memory (Form A for Baseline and Form B for first post-treatment follow-up visit; subsequent follow-up visits will alternate). The NIH Toolbox will not be performed at Month 1-5 follow-up visits if these visits are conducted remotely. |
| Test of Memory Malingering (TOMM) | Secondary Outcome Measure | Baseline, Post aiTBS, and Follow-up | The TOMM is a visual recognition test designed to help distinguish between malingered and true memory impairments (10). Research has found the TOMM to be sensitive to malingering or deliberately reduced effort. Performance is generally preserved in the setting of many neurological impairments including mild cognitive impairment, dementia, aphasia, and TBI. The TOMM will take approximately 25 minutes to complete. |
| Assessment of Blinding | Blinding | First and Final rTMS session | The Assessment of Blinding forms will be completed both by the participant, as well as the TMS operator, at two points, after the first rTMS session and after the final rTMS session in the Randomization phase. The form will use a 1-5 Likert scale. The form takes approximately 5 minutes to complete. |

**References:**

1. Corrigan JD, Bogner J. Initial reliability and validity of the Ohio State University TBI Identification Method. J Head Trauma Rehabil. 2007;22(6):318–29.

2. Bush K, Kivlahan DR, McDonell MB, Fihn SD, Bradley KA, for the Ambulatory Care Quality Improvement Project (ACQUIP). The AUDIT Alcohol Consumption Questions (AUDIT-C): An Effective Brief Screening Test for Problem Drinking. Archives of Internal Medicine. 1998 Sep 14;158(16):1789–95.

3. Sinadinovic K, Wennberg P, Berman AH. Targeting problematic users of illicit drugs with Internet-based screening and brief intervention: a randomized controlled trial. Drug Alcohol Depend. 2012 Nov 1;126(1–2):42–50.

4. Bushnell DM, McCarrier KP, Bush EN, Abraham L, Jamieson C, McDougall F, et al. Symptoms of Major Depressive Disorder Scale: Performance of a Novel Patient-Reported Symptom Measure. Value Health. 2019 Aug;22(8):906–15.

5. Rush AJ, Giles DE, Schlesser MA, Fulton CL, Weissenburger J, Burns C. The Inventory for Depressive Symptomatology (IDS): preliminary findings. Psychiatry Res. 1986 May;18(1):65–87.

6. Blevins CA, Weathers FW, Davis MT, Witte TK, Domino JL. The Posttraumatic Stress Disorder Checklist for DSM-5 (PCL-5): Development and Initial Psychometric Evaluation. J Trauma Stress. 2015 Dec;28(6):489–98.

7. Ehring T, Zetsche U, Weidacker K, Wahl K, Schönfeld S, Ehlers A. The Perseverative Thinking Questionnaire (PTQ): Validation of a content-independent measure of repetitive negative thinking. J Behav Ther Exp Psychiatry. 2011 Jun;42(2):225–32.

8. Tulsky DS, Kisala PA, Victorson D, Carlozzi N, Bushnik T, Sherer M, et al. TBI-QOL: Development and Calibration of Item Banks to Measure Patient Reported Outcomes Following Traumatic Brain Injury. J Head Trauma Rehabil. 2016;31(1):40–51.

9. Weintraub S, Dikmen SS, Heaton RK, Tulsky DS, Zelazo PD, Bauer PJ, et al. Cognition assessment using the NIH Toolbox. Neurology. 2013 Mar 12;80(11 Suppl 3):S54-64.

10. Tombaugh TN. The Test of Memory Malingering (TOMM): Normative data from cognitively intact and cognitively impaired individuals. Psychological Assessment. 1997;9(3):260–8.
