# Supplementary material for: Adaptive trial for the treatment of depressive symptoms associated with concussion using accelerated intermittent theta burst stimulation (ADEPT): rationale, design and methods
Source: Front Neurol. 2025 Jun 13;16:1605157. doi: 10.3389/fneur.2025.1605157 (PMC12202228; doi:10.3389/fneur.2025.1605157)
Supplement: Supplementary file 4 [file Table_4.docx]

**Supplementary Table 4: Secondary Analyses**

| Secondary Analysis | Hypothesis | Statistical Test |
| --- | --- | --- |
| Compare proportion of participants in each condition achieving treatment response (≥50% improvement in MADRS) | The best performing aiTBS arm will lead to a statistically significantly greater number of participants who achieve treatment response as compared to sham stimulation. | Logistic Regression |
| Compare proportion of participants in each condition who achieve remission (final MADRS of ≤10) | The best performing aiTBS arm will lead to a statistically significantly greater number of participants who achieve remission as compared to sham stimulation. | Logistic Regression |
| Compare change in MADRS scores from Baseline to Post-aiTBS in the subgroup of participants who achieve compliance (completing ≥80% of rTMS sessions) | In those who achieve compliance, the best performing aiTBS arm will lead to a statistically significant and clinically meaningful ≥4 points depressive symptom reduction at the Post-aiTBS evaluation as compared to sham stimulation. | Bayesian (analogous to the analysis described above) |
| Compare duration of remission of depressive symptoms over the 6-Month Follow-up phase of the study | Significantly fewer participants in the best performing aiTBS arm will experience a relapse of depressive symptoms and relapses will occur later as compared to sham stimulation. | Log-rank test (survival analysis) |
| Compare change in MADRS scores from Baseline to 6-Month Follow-up | The best performing aiTBS arm will lead to a statistically significant and clinically meaningful ≥4 points depressive symptom reduction at the 6-Month Follow-up as compared to sham stimulation. | Bayesian (analogous to the analysis described above) |
| Assess self-reported depression symptoms as reflected by the IDS-SR and SMDDS | The best performing aiTBS arm will lead to a greater self-reported depressive symptom reduction at both the Post-aiTBS and 6-Month Follow up evaluation as compared to sham stimulation. | Factorial Analysis of Variance (ANOVA) |
| Assess changes in TBI-related symptoms as reflected by the TBI-QOL | The best performing aiTBS arm will lead to a greater reduction in TBI-related symptoms at both the Post-aiTBS and 6-Month Follow up evaluation as compared to sham stimulation. | Factorial Analysis of Variance (ANOVA) |
| Assess changes in PTSD-related symptoms as reflected by the PCL-5 | The best performing aiTBS arm will lead to a greater reduction in PTSD-related symptoms at both the Post-aiTBS and 6-Month Follow up evaluation as compared to sham stimulation. | Factorial Analysis of Variance (ANOVA) |
| Assess changes in RNT as reflected by the PTQ | The best performing aiTBS arm will lead to a greater reduction in RNT at both the Post-aiTBS and 6-Month Follow up evaluation as compared to sham stimulation. | Factorial Analysis of Variance (ANOVA) |
| Assess changes in the NIH Toolbox cognitive battery (for participants with valid effort, as assessed with the TOMM) | The best performing aiTBS arm will lead to a greater improvement in cognitive functioning at both the Post-aiTBS and 6-Month Follow up evaluation as compared to sham stimulation. | Factorial Analysis of Variance (ANOVA) |
| Compare proportion of participants in each condition who achieve compliance (completing ≥80% of rTMS sessions) | All arms will achieve tolerability and acceptability minimum standards with no significant difference in compliance across arms | Logistic Regression |
| Assess changes in rsfMRI connectivity focusing on brain networks relevant to mood regulation | ICT-aiTBS will lead to enhanced functional connectivity in brain networks related to mood regulation at both the Post-aiTBS and 6-Month Follow up evaluation as compared to scalp-based aiTBS, or sham stimulation. | Factorial Analysis of Variance (ANOVA) |
